# Optimizing a Drone Network to Respond to Opioid Overdoses

**DOI:** 10.5811/westjem.59609

**Published:** 2023-08-30

**Authors:** Daniel J. Cox, Jinny J. Ye, Chixiang Zhang, Lee Van Vleet, João R. Nickenig Vissoci, Daniel M. Buckland

**Affiliations:** * Duke University, Department of Emergency Medicine, Durham, North Carolina; † Duke University, Department of Electrical and Computer Engineering, Durham, North Carolina; ‡ Durham County Emergency Medical Services, Durham, North Carolina; § Global Health Institute, Duke University, Durham, North Carolina; ∥ Duke University, Department of Mechanical Engineering and Materials Science, Durham, North Carolina

## Abstract

**Introduction:**

Effective out-of-hospital administration of naloxone in opioid overdoses is dependent on timely arrival of naloxone. Delays in emergency medical services (EMS) response time could potentially be overcome with drones to deliver naloxone efficiently to the scene for bystander use. Our objective was to evaluate a mathematical optimization simulation for geographical placement of drone bases in reducing response time to opioid overdose.

**Methods:**

Using retrospective data from a single EMS system from January 2016–February 2019, we created a geospatial drone-network model based on current technological specifications and potential base locations. Genetic optimization was then used to maximize county coverage by drones and the number of overdoses covered per drone base. From this model, we identified base locations that minimize response time and the number of drone bases required.

**Results:**

In a drone network model with 2,327 opioid overdoses, as the number of modeled drone bases increased the calculated response time decreased. In a geospatially optimized drone network with four drone bases, response time compared to ambulance arrival was reduced by 4 minutes 38 seconds and covered 64.2% of the county.

**Conclusion:**

In our analysis we found that in a mathematical model for geospatial optimization, implementing four drone bases could reduce response time of 9–1–1 calls for opioid overdoses. Therefore, drones could theoretically improve time to naloxone delivery.

## INTRODUCTION

In 2021 over 100,000 people died from opioid overdoses in the United States.^1^ Opioid death rates also increased 10-fold from 2013 to 2019.^2^ Although death from an opioid typically occurs over a few hours,^3^ there may be bystander delays in calling 9–1–1.^4^^–^^6^ The reduction of deaths from opioid overdoses often depends on prompt 9–1–1 response and use of the reversal medication naloxone.

Naloxone is currently available without a prescription in intranasal and intramuscular forms that lay-users can safely administer with little skill. Because of this, bystanders can play a vital role in preventing deaths from opioid overdoses through the administration of naloxone. Despite expanded access and education on naloxone, people who use opioids may not carry this life-saving medication.^7^ Furthermore, time to ambulance arrival, which can impede the provision of naloxone, ranges from 1.7–51 minutes (median 6.9 minutes).^8^ National Fire Protection Agency Standard 1710 establishes target response times for EMS personnel. It states that the goal for response times is five minutes for 90% of dispatched incidents.^9^ Analysis from 2017 suggested that EMS response times averaged 7 minutes in the US, two minutes longer than NFPA 1710.^10^

Unmanned aerial vehicles, or drones, may offer a bridge for faster naloxone delivery while awaiting trained first responders (firefighters, police, or paramedics). Drones have previously been used for medication delivery in rural areas,^7^ disease surveillance and collection of biosamples,^11^ and they have been investigated for use in automatic external defibrillators delivery in cardiac arrest.^12^ Recently, a study by Tukel et al demonstrated that drones have the ability to travel straight-line distances carrying life-saving opioid reversal medications faster than ambulances.^13^ Additionally, a pre-print by Lejune and Ma describes use of a stochastic method to improve response times by 78% in Virginia Beach.^14^

Our objective in this study was to determine whether a mathematical model could be used to optimize the placement of drone bases to reduce response time to opioid overdoses. Given the locations of opioid overdoses, this mathematical model determines the number and location of drone bases to meet any specified reduction in response time. When compared to observed ambulance response times, our optimized network was found to reduce response times to opioid reversal medication delivery by over four minutes.

## METHODS

### Study Setting

The study was conducted in a county in North Carolina with a population of over 300,000 people in 2019 on ≈750 square kilometers of land with an average population density of 434 per square kilometer.^15^ A single EMS agency responds to medical emergencies for this area. In all suspected opioid overdose calls, firefighters and sheriff’s deputies are dispatched along with EMS. However, we only had access to the EMS data for this study. Note that the data does not include response times for law enforcement, firefighters, or other emergency medical responders.

Dispatching ambulances use a hybrid system status management (SSM). The hybrid SSM coordinates responding units based in stations and automatic vehicle location (AVL) to guide dispatch. The AVL ensures that the unit closest to the call is assigned to the response based on GPS. The paramedic service also strategically posts units based on call volume. For example, if all responding units are busy, outlying units will be sent to general geographic areas to provide coverage in uncovered areas during a high-volume period. General study setting information (eg, county demographics, response times) are shown in Table 1. We defined “dispatch time” as the time between when the call came in and when a unit or drone was assigned to that particular incident.

**Table 1.** Summary statistics for Durham County, North Carolina.

County Demographic and Historical Response Data	
Characteristics	
Population (2017)	311,640
Population density (2017), km^2^	935.7
Average annual number of opioid overdoses	743.1
Female gender in overdoses	43%
Average patient age, years	41
Number of paramedic and fire stations	29
Dispatch time, minutes/seconds	
Average	03:01
90th percentile	05:00
Response time, minutes/seconds	
Average	10:46
90th percentile	17:00

### Study Design

Using a retrospective cross-sectional design, we used data in prospectively collected electronic health records as part of routine care. Our overall design was inspired by Boutilier et al,^16^ which mathematically optimized drone networks for automatic external defibrillators (AED) for out-of-hospital cardiac arrest in Toronto, Canada. Their method optimized by a threshold response time, determining how many drones at each prospective base would be required to reduce response times by one, two, and three minutes. We furthered this analysis method by not holding the number of bases constant and optimized the number of drones and bases needed to produce the greatest coverage area and produce the greatest decrease in response time per drone.

## DATA SOURCES

### Opioid Overdose Episodes

We included in this study all dispatches for suspected opioid overdoses in the service area from January 1, 2016–February 17, 2019 (Emergency Medical Dispatch card number 23 or naloxone administration during the call) regardless of call priority). Locations of these dispatches were automatically geocoded (converting a text-based address to geographic coordinates) based on addresses obtained during dispatch calls.

### Candidate Base Locations

For the drone network, all fire, paramedic, and EMS administrative buildings were considered potential drone bases. The addresses of each station were obtained from the local paramedic service and geocoded into Universal Transverse Mercator (GIS Geography, Redlands, CA) coordinates. We used 29 such candidate stations in our analysis. Drone bases were selected among these candidate stations.

### Drone Specifications

Drone parameters for our model were chosen to reflect current, cost-effective drone capabilities. We set the maximum speed as 18 kilometers per hour and a maximum distance of 7 kilometers on one battery life. We chose more conservative metrics than Boutilier et al because naloxone will require less power to transport compared to an AED. However, Tukel et al used drones that could reach speeds of 94 kilometers per hour, which would likely further improve results from this study. These drones had the ability to carry up to 1.4 kilograms in payload, which should easily allow for naloxone to be transported. Because there is no previously standardized launch time, we assumed drone lift off and landing would each take 60 seconds. We did not account for time for lay-users to reach the drone, remove the medication, and return to the patient’s side, as these calculations have not been established. Ambulance response time also does not account for the time interval between EMS arrival on scene to arrival to patient.

Each drone’s geographical catchment area was set to a 3.2 kilometer-(two mile) radius for the drone’s ability to fly to and from the scene. We also accounted for wind speed and rain on the days of overdose incidents using hourly North Carolina climate data in the service area.^17^ Assuming the drone could withstand a level 5 wind (wind speeds of 19–24 miles per hour), we set 19 miles per hour as the threshold speed for drones to work normally. For most drones, it is not recommended to operate in rainy conditions. We assumed that drone service would pause during these times (precipitation >0).

## ANALYSIS

### Measurements

**Figure 1.**
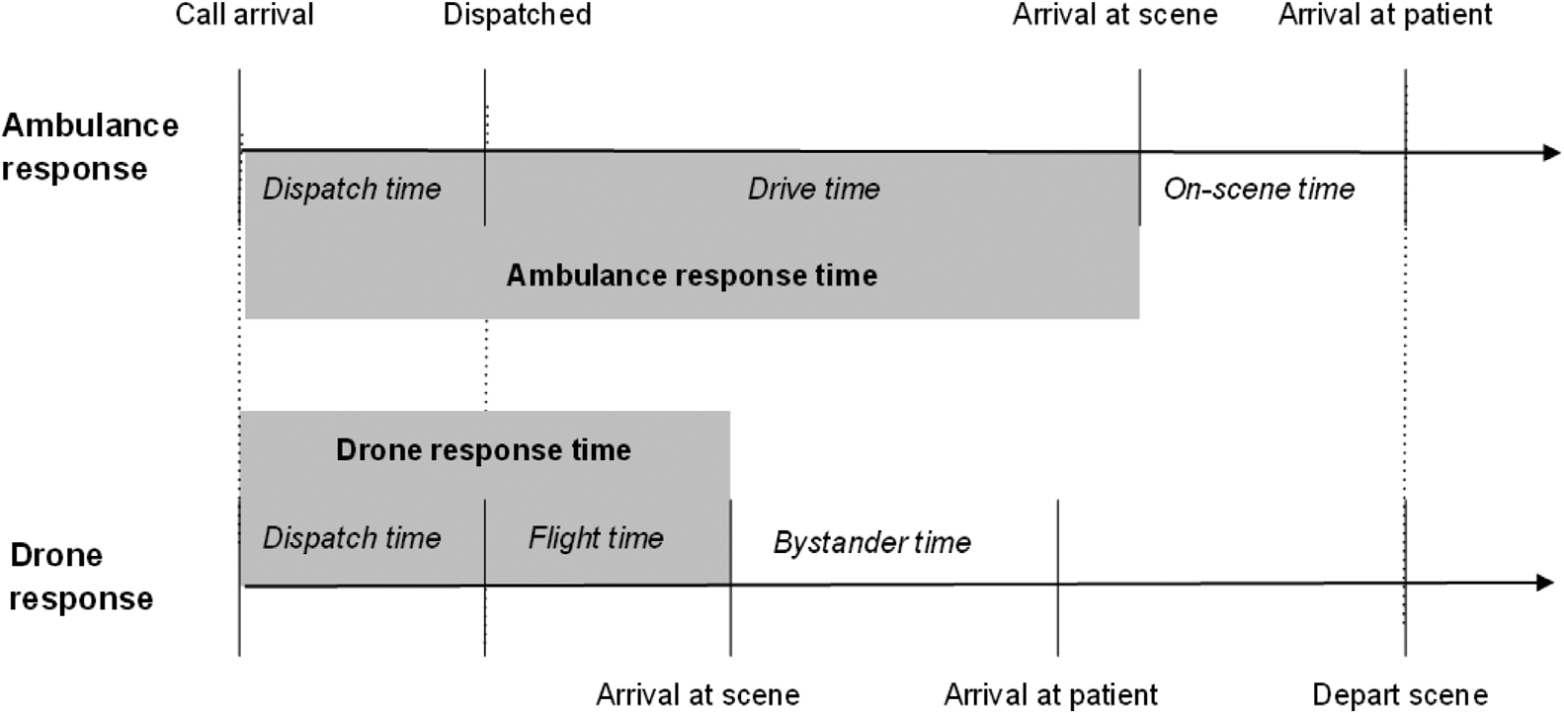
Ambulance and drone timelines adapted from Boutilier et al 2017.^16^ The objective of the drone is to get naloxone to the patient as early as possible. If the ambulance arrives first, the drone on-scene time will be zero. If the drone arrives first, naloxone can be given while the ambulance is in route.

Our primary outcomes were ambulance and drone response times as depicted in Figure 1. For our dataset, we defined ambulance response time as the duration between EMS assignment and arrival at scene as documented in EMS records. Drone response time was considered time between drone assignment and arrival at scene. These definitions were equivalent to those used by Boutilier et al. Drones would be collected from the scene when an ambulance arrives. Time between arrival on scene and arrival to the patient was not calculated for either drone or ambulance response times. We calculated drone response times using a genetic optimization model and technological specifications of current drones as described above. Additional time was added to the calculated flight time to account for dispatching the drone and allowing it to land after arriving on the field site. This additional time produced a very conservative estimate of the total response time for each drone flight. We excluded incidents with missing data on scene location and response time.

### Genetic Optimization

Genetic optimization is an iterative process inspired by natural selection in which the properties of a potential solution to a problem are changed with each iteration. After each iteration (generation), the performance (fitness) is assessed for each potential solution. The subsequent generation of solutions is then determined based on the composition of the current highest performing solutions. In biological terms, the best solutions in each generation serve as the parents for the next set of potential solutions. This process continues until a solution meets a set of minimum criteria.^18^^,^^19^

Our goal was to determine the optimal number and spatial geographic distribution of drone bases that would reduce naloxone distribution times to opioid overdoses. To accomplish this, we first used locations of overdose incidents, number of drone bases, and locations of drone bases to create drone base networks. These networks each had a unique combination of base locations, number of bases, coverage rate (percentage of incidents covered by drone bases), and coverage density (number of incidents covered by a drone base). Given the prespecified number of bases from 0–29, the genetic optimization model determines the geographical locations of drones that maximizes coverage rate and density (in mathematical terms, if entropy converged). Maximizing coverage rate and density did not account for rural and urban differences in response times. Our algorithm sought to improve the overall distribution of response times, regardless of location. For example, base locations for a two-drone network may be completely different than a three-drone network rather than simply adding another drone location. The genetic optimizer was considered successful for drone network optimization if entropy converged. We conducted analyses with Python 3.6.8 (Python Software Foundation, Wilmington, DE) on Jupyter Notebook and ArcGIS Desktop 10.5.1 (Esri).

### Observed and Estimated Response Time

We found that the drone networks improved average response time for each added drone base. For each reduction in response time, we identified the locations of these drone bases chosen from candidate EMS/fire stations. We calculated the distributions of the observed response times and estimated drone response times for each incident. A graph of response time and number of drone bases were plotted. We chose the operating point, or the point closest to the graph origin, as the optimum number of drone bases that would minimizs the response time and number of bases.

We performed the test of significance with a Student *t*-test by proposing the null hypothesis *H*_0_: the average (or expected) response time of historical, optimized, and fully deployed delivery network is different from each other. We calculated the *P*-value for each paired combination of historical, optimized and fully deployed delivery networks. *P*-values less than 0.05 were considered significant.

### Ethics Statement

The Duke University Institutional Review Board determined this study to be exempt (Pro00101461).

## RESULTS

### Characteristics of Study Subjects and Climate

From 2016 to early 2019, 2,634 calls were dispatched for suspected opioid overdose/poisoning. After eliminating duplicate incidents with unique responding units, we found 2,327 distinct incidents of suspected overdose/poisoning or dispatch calls in which naloxone was used. We excluded 303 cases in which response time data were missing, three cases in which response times were greater than 40 minutes and assumed to be inaccurate documentation, and one case that was geocoded incorrectly, resulting in a total of 2020 final encounters. Summary characteristics for opioid overdoses are shown in Table 1. Of the included cases of opioid overdoses 43% involved females, and the average age was 41 years old. Dispatch time took an average of 3 minutes 1 second, and response time took an average of 10 minutes 46 seconds. Regarding climate, no overdoses occurred when winds exceeded 19 miles per hour, while 147 incidents occurred during rain. We did not exclude in the genetic algorithm the incidents that occurred in the rain.

### Main Results

The entropy of the genetic optimization converged after 60 generations, meaning the drone base network was able to be optimized. The change in response time and coverage rate with each added drone base is shown in Table 2. Response time and coverage rate consistently improved with increasing number of drone bases up to the 29 available locations. For example, having only one drone base reduced response time by 2 minutes 24 seconds with a 36% coverage rate. But at full deployment of 29 bases, response time was reduced by 8 minutes 12 seconds and covered 97.8% of incidents.

**Table 2.** Drone network characteristics for the response time improvement in opioid overdoses.

Number of drone bases	Improvement in response time, mm:ss	Coverage rate %
0	00:00	0.0
1	02:24	35.8
2	03:28	50.5
3	03:57	56.3
4	04:38	64.2
5	05:03	70.9
6	05:24	73.0
7	05:47	76.8
8	06:05	79.9
9	06:19	82.1
10	06:38	86.5
11	06:51	86.9
12	07:02	90.3
13	07:08	91.3
14	07:18	91.4
15	07:25	91.3
16	07:31	92.7
17	07:34	92.7

**Figure 2.**
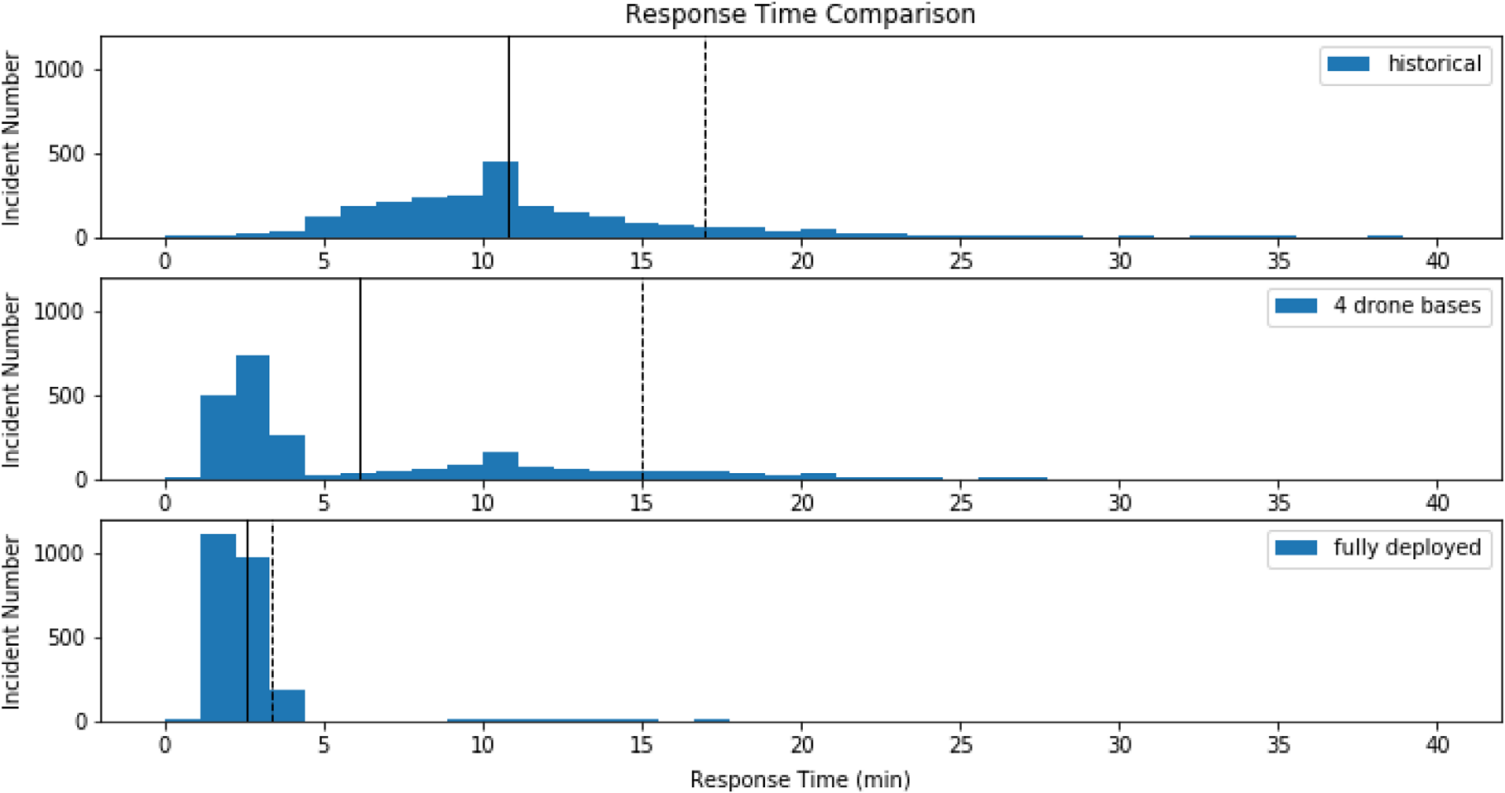
Comparison of response time distributions. The top row demonstrates the distribution of historical ambulance response times. The second row shows the distribution of estimated response time corresponding to the four-base drone network. The third row shows the distribution of estimated response time if all candidate bases were chosen as the part of the drone network. (Drone bases are fully deployed.) Solid vertical lines demonstrate the mean response time. Dashed vertical lines represent the 90th percentile in response time.

**Figure 3.**
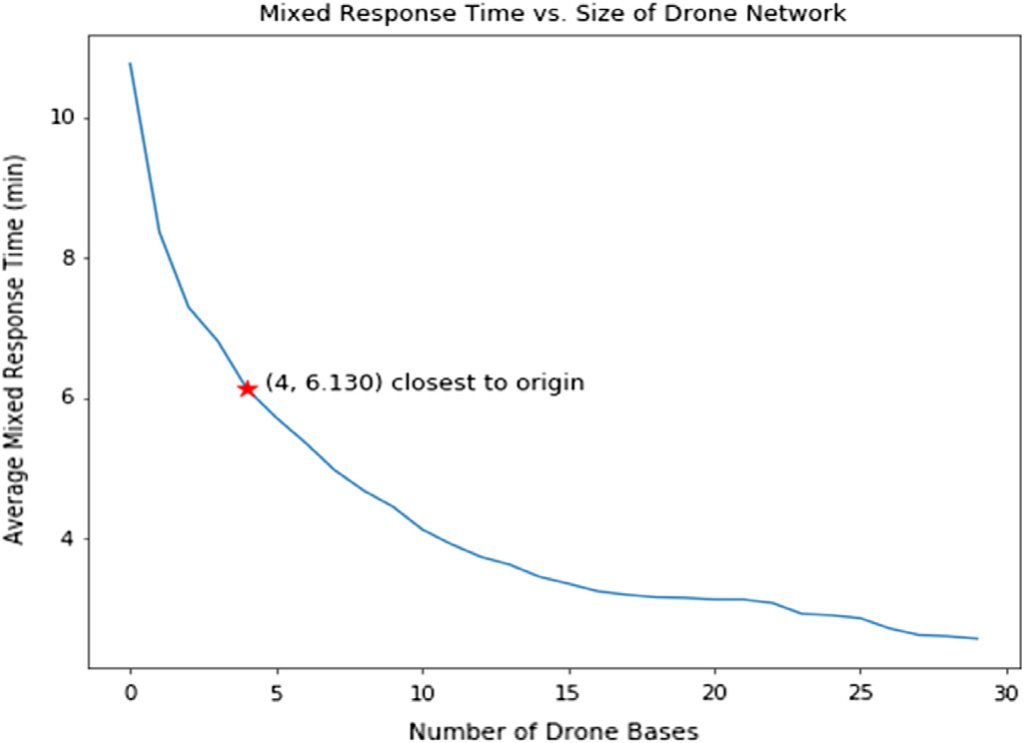
Average response time given number of drone bases. This figure demonstrates the relationship between the number of drone bases and response time. The red star represents the point closest to the origin (x-axis must be a whole number). This point corresponds to the most efficient use of drone resources.

**Figure 4.**
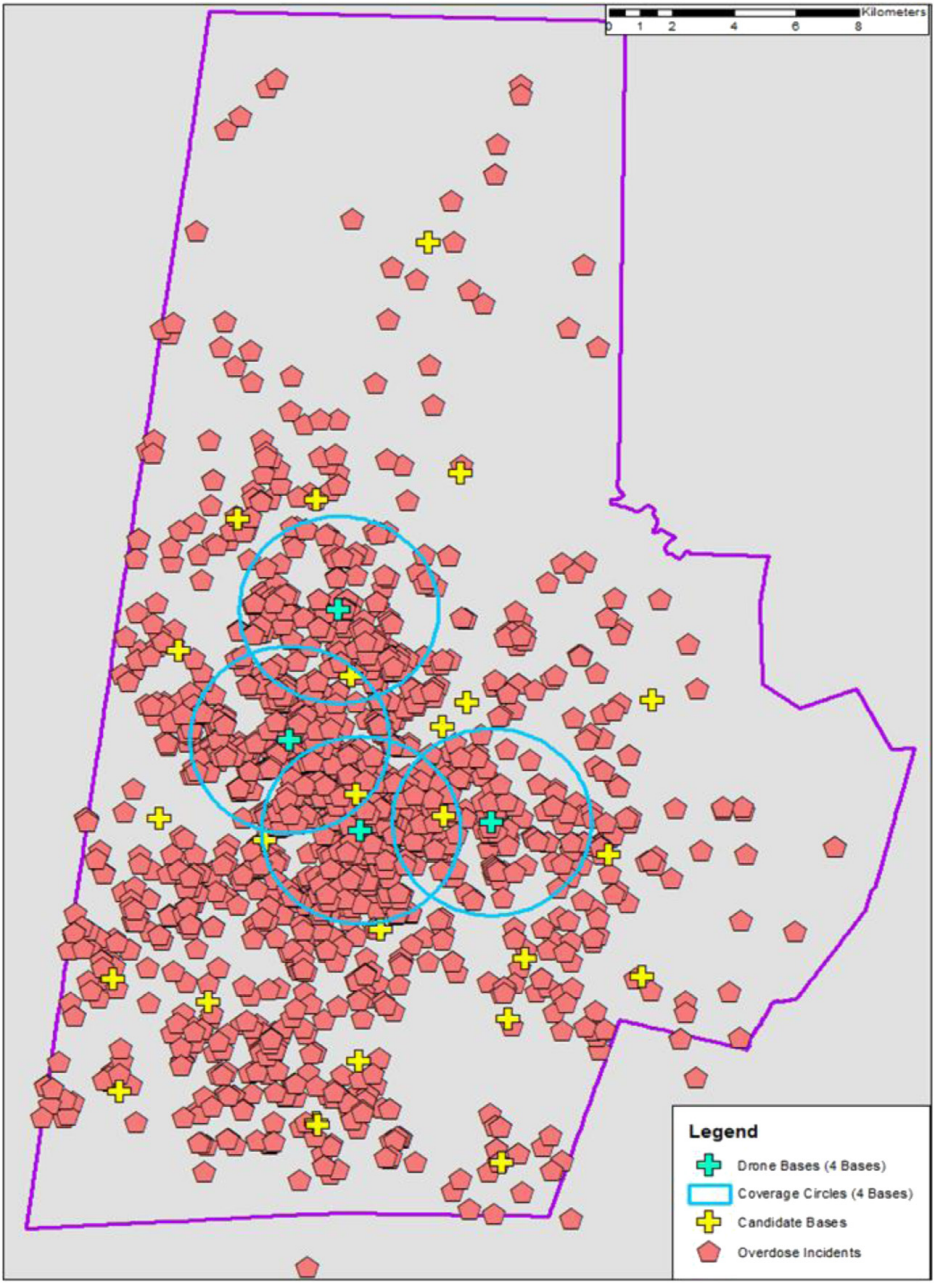
**Historical opioid overdoses, paramedic station locations, and optimal drone network.** This county map demonstrates locations of historical opioid overdoses (red pentagons). Additionally, it shows the locations of all candidate drone bases (+ signs) and the optimized drone network (blue + signs) and its coverage area (area inside blue circles).

The distribution of response times also narrowed with an increase in drone bases (Figure 2). As more drone bases were introduced into the network, the average and range of estimated response times are markedly decreased compared to historical ambulance response times. Because response time cannot be improved without increasing the number of drone bases, we chose a drone network that minimized response time and number of bases, thereby maximizing resource utilization. This ideal network, shown as the point closest to origin (Figure 3), was four drone bases. These optimal candidate bases improved average response time by 4 minutes and 38 seconds with a coverage rate of 64.2%. These bases are in areas with more opioid overdoses (ie, urban areas), leaving rural areas largely uncovered by drones due to constraints on flight time and distance (Figure 4).

## DISCUSSION

In this study we evaluated the potential benefit of using drones to improve time to naloxone delivery in suspected opioid overdoses. Our analysis found that a mathematical model can optimize the location and number of bases to reduce naloxone delivery time compared to historical ambulance response times. We found that drones not only improve the average time of naloxone delivery on scene (Table 2), but also reduce the entire response time distribution (Figure 2).

Drone delivery of naloxone has the potential to reduce the time to naloxone administration and theoretically reduce mortality from opioid overdoses. Our conservative model demonstrates that drones could be used to deliver naloxone ahead of ambulances. Compared to ambulances, drones may overcome challenges and delays in reaching private locations.^8^ In simulated out-of-hospital cardiac arrests, drones have reduced response time by 16 minutes compared to EMS.^12^

In our optimized model, the locations of our drone bases leave the most rural locations to be covered only by ambulances. Drone networks can cover rural areas. However, the number of drones needed to cover rural areas would likely not be economical at the current range of Federal Aviation Administration (FAA)-rated commercial drones unless there was a cluster of opioid overdoses where drones could be stationed nearby. The genetic algorithm did not directly account for rural/urban differences. Instead, it focused on covering the maximum number of overdoses, of which the vast majority occur in urban areas. Thus, our drone network maximizes our chances of reducing mortality in opioid overdoses, especially during times of increased traffic and call volume which can prolong ambulance response times.

Although all states currently have some form of naloxone access laws, studies suggest that Blacks are less likely to have access to naloxone.^20^^–^^22^ With Blacks making up 36.9% of the population in the service area, a drone network has the potential to provide this lifesaving treatment to those who may not have equitable access.

## LIMITATIONS

Because the model depended on the estimated incidence of opioid overdoses in each catchment area, bases in high-call volume areas will have more drone busy time and require more drones. However, the remaining parameters were more conservative, likely leading to an overestimate in drone network size. We assumed only one drone per base, whereas in reality multiple drones could be deployed from one base, decreasing drone busy time. Drone resources may also be overestimated because we included dispatches for opioid overdoses that did not receive naloxone. This small fraction of cases may be ruled out for future drone deployment. Additionally, drone technology is progressing rapidly. With our conservative drone specifications, response times are likely an overestimate. Furthermore, potential candidate bases locations such as hospitals and clinics were not included in the model. Having these additional locations could further reduce drone response times.

We additionally used Euclidean distance to set our coverage area and did not account for FAA regulations on drone flight paths, which may require a slightly more circuitous path to bystanders than anticipated by this model. Further, difficult weather conditions (eg, wind and rain) may limit the ability to fly drones based on technological capabilities. Reassuringly, the number of overdose incidents during these conditions were minimal. Thus, we expect real-life implementation of drones to not be majorly impacted by weather.

Lastly, the data we used was incomplete because we did not have access to data from law enforcement or fire departments. This may have led to an overestimation of historical ambulance response times. In addition, data was based on emergency medical dispatch (EMD) impression of the chief complaint as a drug overdose. Calls in which EMS impression was opioid overdose, but EMD impression was not, may have been missed. We attempted to account for this difference by including all calls in which naloxone was administered, regardless of impression. Other opioid overdose cases may have been unaccounted for due to data exclusion based on incomplete or suspected inaccurate documentation on response times. However, because we excluded only 11.5% of cases based on documentation, the impact of missing data is likely small.

## CONCLUSION

We have established that a drone network mathematically optimized in location and number of drone bases would potentially reduce time to naloxone delivery in opioid overdoses compared to historical ambulance response times. Our next steps will be to closely examine the feasibility of implementing such a drone network by measuring GPS signals, confirming flight paths, and completing live drone tests. As drone technology improves and cost decreases, future utilization of drone networks could help temporize many emergency medical situations until EMS arrives.
